# T4 Phage and Its Head Surface Proteins Do Not Stimulate Inflammatory Mediator Production

**DOI:** 10.1371/journal.pone.0071036

**Published:** 2013-08-16

**Authors:** Paulina Miernikiewicz, Krystyna Dąbrowska, Agnieszka Piotrowicz, Barbara Owczarek, Justyna Wojas-Turek, Jagoda Kicielińska, Joanna Rossowska, Elżbieta Pajtasz-Piasecka, Katarzyna Hodyra, Katarzyna Macegoniuk, Kamila Rzewucka, Agnieszka Kopciuch, Tomasz Majka, Andrey Letarov, Eugene Kulikov, Henryk Maciejewski, Andrzej Górski

**Affiliations:** 1 Institute of Immunology and Experimental Therapy, Polish Academy of Sciences, Wroclaw, Poland,; 2 Winogradsky Institute of Microbiology, Russian Academy of Sciences, Moscow, Russia,; 3 Institute of Computer Engineering, Control and Robotics, Wroclaw University of Technology, Wroclaw, Poland,; 4 Department of Clinical Immunology, Transplantation Institute, Medical University of Warsaw, Warsaw, Poland; Agency for Science, Technology and Research - Singapore Immunology Network, Singapore

## Abstract

Viruses are potent activators of the signal pathways leading to increased cytokine or ROS production. The effects exerted on the immune system are usually mediated by viral proteins. Complementary to the progress in phage therapy practice, advancement of knowledge about the influence of bacteriophages on mammalian immunity is necessary. Particularly, the potential ability of phage proteins to act like other viral stimulators of the immune system may have strong practical implications for the safety and efficacy of bacteriophage therapy. Here we present studies on the effect of T4 phage and its head proteins on production of inflammatory mediators and inflammation-related factors: IL-1α, IL-1β, IL-2, IL-6, IL-10, IL-12 p40/p70, IFN-γ, TNF-α, MCP-1, MIG, RANTES, GCSF, GM-CSF and reactive oxygen species (ROS). Plasma cytokine profiles in an *in vivo* mouse model and in human blood cells treated with gp23*, gp24*, Hoc and Soc were evaluated by cytokine antibody arrays. Cytokine production and expression of CD40, CD80, CD86 and MHC class II molecules were also investigated in mouse bone marrow-derived dendritic cells treated with whole T4 phage particle or the same capsid proteins. The influence of T4 and gp23*, gp24*, Hoc and Soc on reactive oxygen species generation was examined in blood cells using luminol-dependent chemiluminescence assay. In all performed assays, the T4 bacteriophage and its capsid proteins gp23*, gp24*, Hoc and Soc did not affect production of inflammatory-related cytokines or ROS. These observations are of importance for any medical or veterinary application of bacteriophages.

## Introduction

Viruses and their components are potent activators of the signal pathways leading to increased cytokine and chemokine production in human and in animals. The effects exerted on the immune system are usually mediated by viral proteins, which stimulate cytokine and/or ROS production in immune cells [1]. There are many examples of such proteins, that also after recombinant expression and purification maintained their pro-inflammatory activity, giving insight into mechanisms of general effect of viruses on the immune system. Glycoprotein gp350 and latent membrane protein 1 (LMP-1) from Epstein-Barr virus are viral proteins giving rise to strong production of interleukin 1 beta (IL-1β), tumor necrosis factor alpha (TNF-1α), IL-6, IL-10 or IL-8 [2]. Also in the course of avian influenza A (H5N1), a virus causing severe disease in humans, hypercytokinemia is a common phenomenon. Among differentiated subtypes of influenza, H5N1 virus indicated the strongest inflammatory cytokine and chemokine production. Its protein NS1 stimulates production of interferon gamma-induced protein 10 (IP-10), monocyte chemotactic protein-1 (MCP-1), monokine induced by gamma interferon (MIG), IL-8, IL-10, IL-6, and interferon gamma (IFN-γ) [3], [4].

Excessive reactive oxygen species (ROS) formation is another potentially harmful effect of the virus activity [5]. For example, core protein of hepatitis C virus (HCV) targets mitochondria and increases ROS generation [6], [7].

Bacteria can also be a target for viruses. However, practical implications of this phenomenon for medicine are different to those of human or animal viruses. Bacterial viruses (bacteriophages, phages) may offer an alternative antimicrobial treatment since the rising number of resistant bacteria has become a worldwide medical problem. Phage ability to attack and kill pathogens was exploited immediately after the discovery of bacteriophages (1915 or 1917) [8]. Phages were applied in anti-bacterial therapy, but the introduction of antibiotics pushed this technology aside. Nowadays, studies on new antimicrobial drugs have been intensified due to increasing resistance of bacteria. Efficacy of phage therapy has been confirmed in various bacterial infections caused by, e.g. methicillin-resistant *Staphylococcus aureus* (MRSA) [9]–[11], *Pseudomonas aeruginosa* [12], [13], and *Escherichia coli*, in a number of research centers [14], [15].

Complementary to the progress in phage therapy practice, advancement of knowledge about the influence of bacteriophages on the mammalian immune system is necessary. Previous studies of bacteriophage interactions with the immune system suggest that at least some phages may exert immunomodulating effects in mammals [16]–[18]. Although some preliminary data on phage – mediated immunobiological activities are available, the exact mechanisms of those interactions remain obscure and require further studies [19]. Particularly, there are no data on the potential ability of phage proteins to act like other viral stimulators of the immune system. Their likely impact on inflammatory mediators may have strong practical implications for safety and efficacy of bacteriophage therapy. Here we present studies on the effect of T4 phage proteins on production of inflammatory mediators using five immunological models *in vitro* and *in vivo*.

Major capsid proteins (gp23*), head vertex protein (gp24*), highly immunogenic outer capsid protein (Hoc) and small outer capsid protein (Soc) (details of T4 phage capsid structure have recently been described and reviewed [20]–[23]). These four structural proteins form the majority of the T4 surface and are strongly exposed to the environment. They also represent the bulk of the total capsid protein contents. Therefore they are possible effective stimulators of immunological processes and were employed for these studies of phage proteins' immunological reactivity.

## Results

### Plasma cytokine profiles in mouse model and human blood treated with T4 phage head proteins

**Figure 1:**
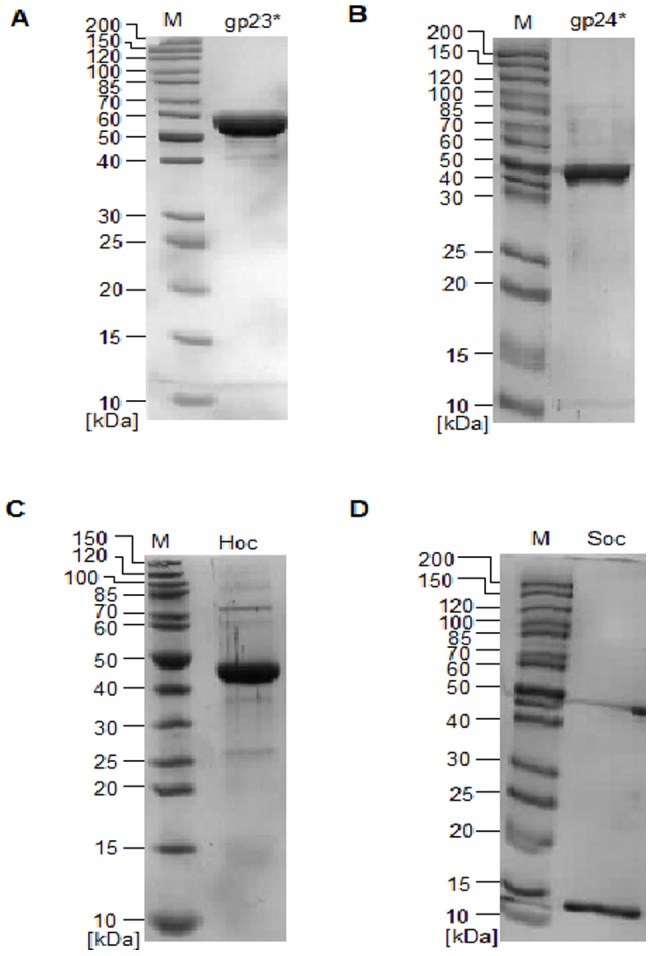
SDS-PAGE of T4 phage head protein preparations. (A) gp23*, (B) gp24*, (C) Hoc and (D) Soc, M – molecular weight marker.

To determine the influence of highly purified T4 phage head proteins (Table 1, Fig. 1) on cytokine production, comparative throughput mouse or human antibody arrays were used. We examined expression of 62 murine and 80 human proteins (including cytokines, chemokines, growth and differentiation factors) (Table 2, Table 3). Out of these, 13 (mouse) or 11 (human) cytokines which are particularly involved in the inflammatory process were selected for statistical analysis: IL-1α, IL-1β, IL-2, IL-6 (mouse only), IL-10 (mouse only), IL-12 p40/p70, IFN-γ, TNF-α, MCP-1, MIG, RANTES, granulocyte colony-stimulating factor (GCSF), and GM-CSF.

**Table 1:** Biochemical characteristics of three exemplary phage protein preparations.

Phage protein	Concentration [mg/ml]	LPS activity [EU/ml]
gp23*	0,656	3,45
	0,454	1,71
	0,821	8,07
gp24*	1,284	7,7
	0,941	5,5
	0,680	1,9
Hoc	2,629	2,8
	2,487	0,48
	2,665	0,2
Soc	0,500	0,017
	0,890	0,067
	0,498	3,2

**Table 2:** Mouse Cytokine Antibody Array map.

Pos	Pos	Neg	Neg	Blank	Axl	BLC	CD30L	CD30T	CD40	CRG-2	CTACK	CXCL-16	Eotaxin
Pos	Pos	Neg	Neg	Blank	Axl	BLC	CD30L	CD30T	CD40	CRG-2	CTACK	CXCL-16	Eotaxin
Eotaxin-2	Fas Ligand	Fracktalkine	GCSF	GM-CSF	IFN-γ	IGFBP-3	IGFBP-5	IGFBP-6	IL-1α	IL-1β	IL-2	IL-3	IL-3Rb
Eotaxin-2	Fas Ligand	Fracktalkine	GCSF	GM-CSF	IFN-γ	IGFBP-3	IGFBP-5	IGFBP-6	IL-1α	IL-1β	IL-2	IL-3	IL-3Rb
IL-4	IL-5	IL-6	IL-9	IL-10	IL-12p40/p70	IL-12p70	IL-13	IL-17	KC	Leptin R	Leptin	LIX	L-Selectin
IL-4	IL-5	IL-6	IL-9	IL-10	IL-12p40/p70	IL-12p70	IL-13	IL-17	KC	Leptin R	Leptin	LIX	L-Selectin
Lymphotactin	MCP-1	MCP-5	M-CSF	MIG	MIP-1α	MIP-1γ	MIP-2	MIP-3β	MIP-3α	PF-4	P-Selectin	RANTES	SCF
Lymphotactin	MCP-1	MCP-5	M-CSF	MIG	MIP-1α	MIP-1γ	MIP-2	MIP-3β	MIP-3α	PF-4	P-Selectin	RANTES	SCF
SDF-1α	TARC	TCA-3	TECK	TIMP-1	TNF-α	sTNF RI	sTNF RII	TPO	VCAM-1	VEGF	Blank	Blank	Pos
SDF-1α	TARC	TCA-3	TECK	TIMP-1	TNF-α	sTNF RI	sTNF RII	TPO	VCAM-1	VEGF	Blank	Blank	Pos

**Table 3:** Human Cytokine Antibody Array map.

Pos	Pos	Pos	Pos	Neg	Neg	ENA-78	GCSF	GM-CSF	GRO	GROα
I-309	IL-1α	IL-1β	IL-2	IL-3	IL-4	IL-5	IL-6	IL-7	IL-8	IL-10
IL-12 p40p70	IL-13	IL-15	IFN-γ	MCP-1	MCP-2	MCP-3	MCSF	MDC	MIG	MIP-1β
MIP-1δ	RANTES	SCF	SDF-1	TARC	TGF-β1	TNF-α	TNF-β	EGF	IGF-I	Angiogenin
Oncostatin M	Thrombopoietin	VEGF	PDGF-BB	Leptin	BDNF	BLC	Ck β 8-1	Eotaxin	Eotaxin-2	Eotaxin-3
FGF-4	FGF -6	FGF -7	FGF -9	Flt-3 Ligand	Fractalkine	GCP-2	GDNF	HGF	IGFBP-1	IGFBP-2
IGFBP-3	IGFBP-4	IL-16	IP-10	LIF	LIGHT	MCP-4	MIF	MIP-3α	NAP-2	NT-3
NT-4	Osteoprotegerin	PARC	PIGF	TGF-β2	TGF-β3	TIMP-1	TIMP-2	Pos	Pos	Pos

**Figure 2:**
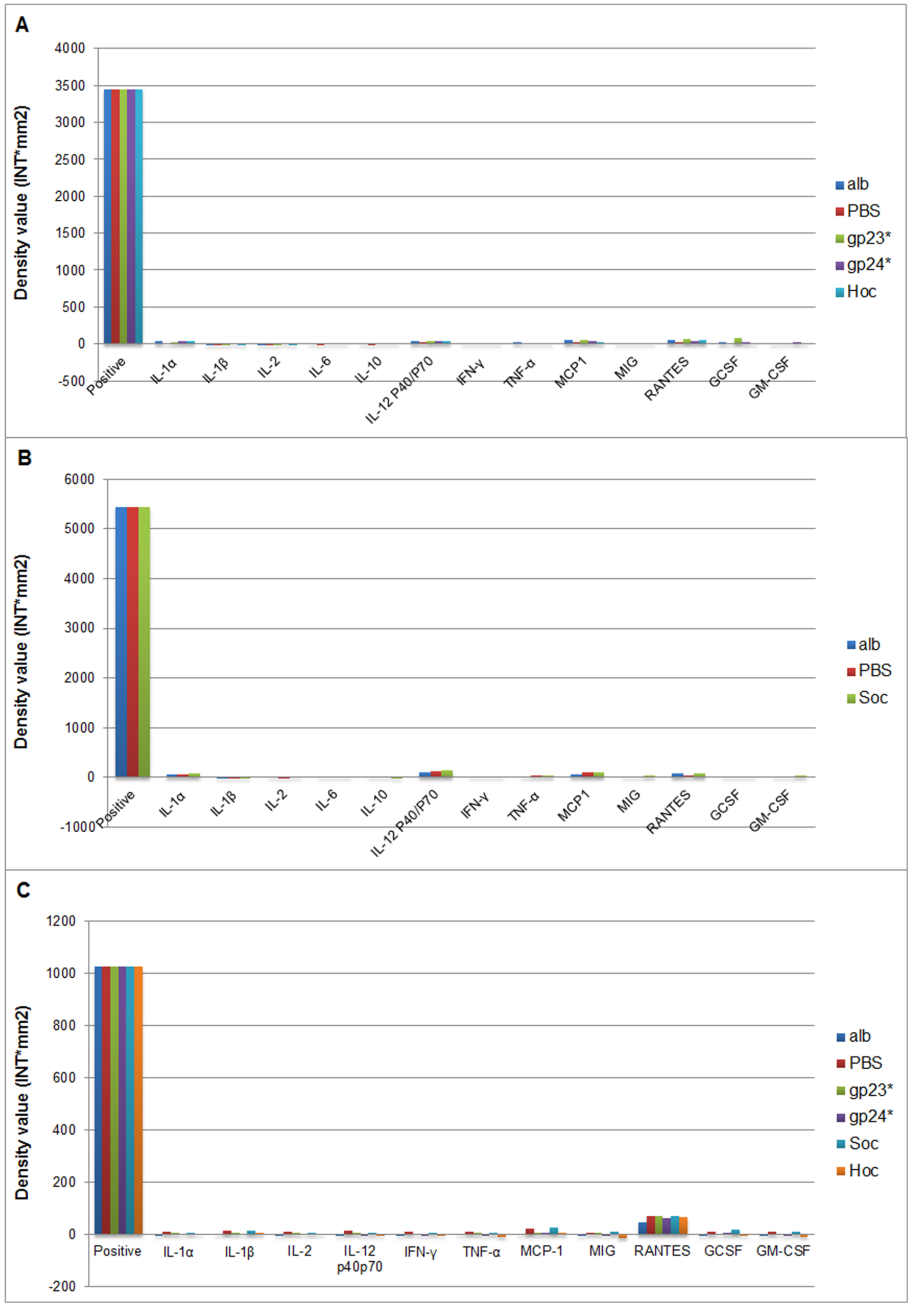
Cytokine profiles in mice and in human blood treated with phage proteins. (A) Cytokine profiles in mice stimulated with gp23*, gp24*, Hoc, PBS, albumin (alb), (B) Cytokine profiles in mice stimulated with Soc, PBS, albumin (alb), (C) Cytokine profiles in human whole blood stimulated with gp23*, gp24*, Hoc, Soc, PBS, albumin (alb).

Mice were injected intraperitoneally with highly purified protein preparations gp23*, gp24*, Hoc, and Soc. Human whole blood, obtained from healthy donors, was incubated with highly purified gp23*, gp24*, Hoc, and Soc. In both cases, PBS and albumin served as negative control groups. For technical reasons, Soc was analyzed in mice in a separate test with the same controls (Fig. 2A: gp23*, gp24*, Hoc, Fig. 2B: Soc). Inflammatory cytokine levels were analyzed and compared to positive controls (within the array layout, according to the manufacturer).

In all cases, cytokine signals in mice treated or human blood incubated with phage proteins were much lower than those of positive controls (Fig. 2). We also did not observe any significant difference in comparison to the negative controls (Fig. 2). Thus, highly purified gp23*, gp24*, Hoc or Soc did not cause increased expression of investigated cytokines *in vivo* in mice or *in vitro* in human blood.

### Cytokine production by dendritic cells treated with T4 phage and its head proteins

To extend the studies on the ability of phage proteins (gp23*, gp24*, Hoc or Soc) to stimulate cytokine production, mouse bone marrow-derived dendritic cells (BM-DCs) were used. Cells treated with 300 EU/ml lipopolysaccharide of *E. coli* (LPS) served as a positive control, whereas cells non-stimulated or albumin-stimulated were negative controls. Preparation of T4 phage (Table 4) was used to complete this comparison. Cell culture supernatants were estimated by ELISA for following cytokines IL-6, TNF-α, IL-10 and IL-12.

**Table 4:** Characteristics of three exemplary T4 phage preparations.

T4 phage preparations	Concentration [pfu/ml]	LPS activity [EU/ml]
1.	2,14×10^10^	2,95
2.	6,75×10^10^	3,00
3.	1×10^10^	1,375

**Figure 3:**
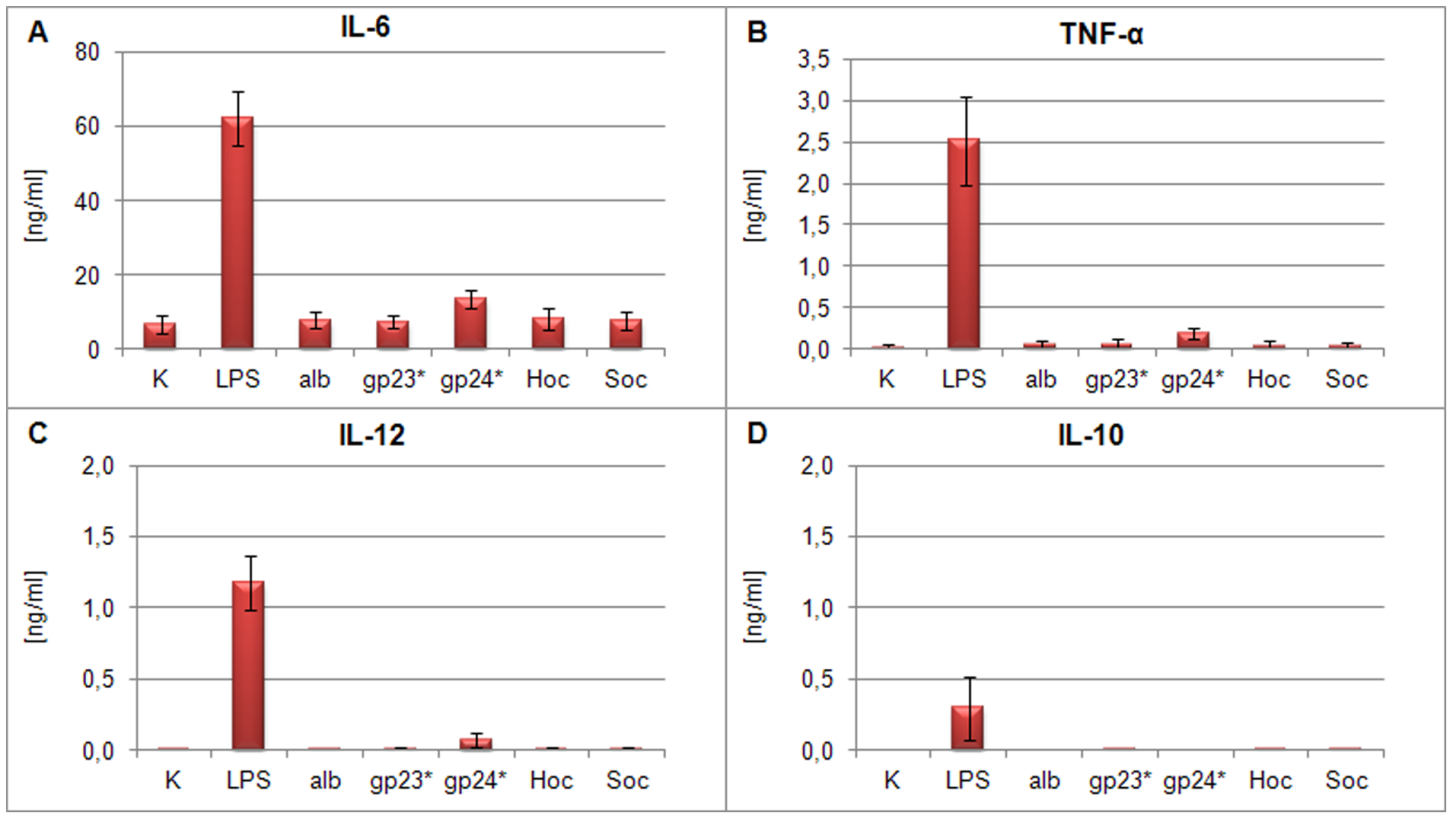
Cytokine production by BM-DCs stimulated with phage proteins. The concentrations of (A) IL-6, (B) TNF-α, (C) IL-12 and (D) IL-10 were determined by ELISA assay in supernatants collected after 24 h stimulation of 1×10^6^ DCs/ml. The data present the means of 3 independent experiments. K – control, alb – albumin.

**Figure 4:**
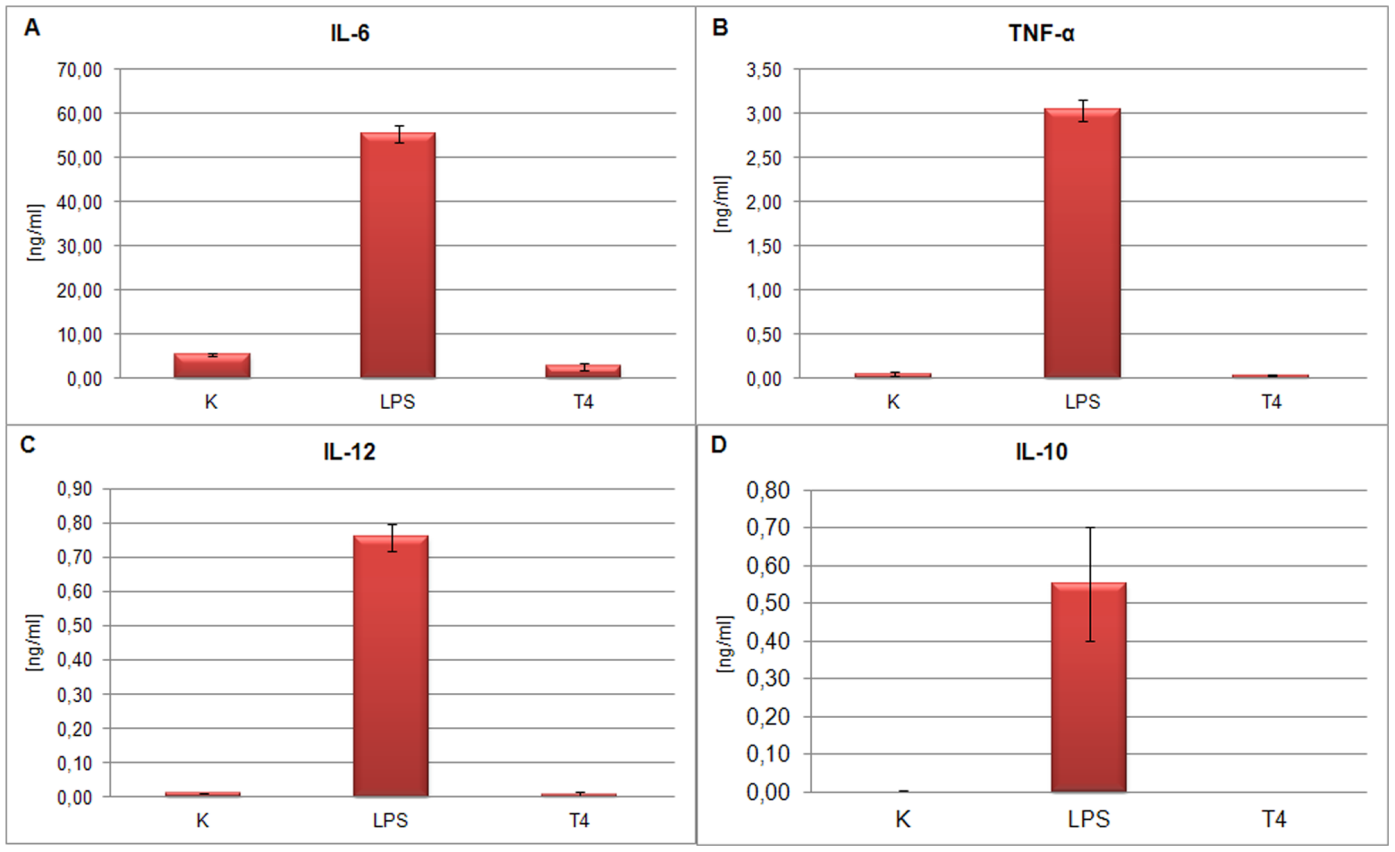
Cytokine production by BM-DCs stimulated with T4 phage. The concentrations of (A) IL-6, (B) TNF-a, (C) IL-12 and (D) IL-10 were determined by ELISA assay in supernatants collected after 24 h stimulation of 1×10^6^ DCs/ml. The data present the means of 3 independent experiments. K – control, alb – albumin.

The level of cytokine production induced by 10 μg/ml of each phage protein as well as by 5×10^8^ pfu/ml of T4 phage was similar to that obtained for the negative control groups. IL-6 production by BM-DCs stimulated with LPS increased up to 59.57±6.64 ng/ml in culture supernatants. By contrast, IL-6 concentration after stimulation with gp23* was 5.95±2,32 ng/ml, with gp24* 10.09±5.33 ng/ml (insignificant in comparison to negative controls), with Hoc 6.59±3.44 ng/ml and with Soc 6.09±2.78 ng/ml. In the negative control cultures similar concentrations of IL-6 were observed: the culture with albumin resulted in 6.55±2.62 ng/ml and with non-stimulated cells amounted to 5.37±3.08 ng/ml (Fig. 3A). T4 phage treatement of BM-DCs also resulted in low production of IL-6 (2.80±0.88 ng/ml). In the case of other cytokines, a similar tendency was observed (Fig. 3B, 3C, 3D): gp23*, gp24*, Hoc, Soc and T4 (Figure 4) did not induce significant production of TNF-α, IL-12, or IL-10.

### The effect of T4 phage and its head proteins on dendritic cell differentiation

Mouse bone marrow-derived cells were also used for the studies on the effect of T4 phage preparations and its capsid proteins on dendritic cell differentiation since potential activation of these cells usually results in changes of their surface antigens. Expression of CD40, CD80, CD86 and MHC class II were compared in cells cultured with whole T4 phage particle as well as its proteins gp23*, gp24*, Hoc, and Soc. Albumin and PBS served as negative controls, while LPS (300 EU/ml) served as a positive control.

**Figure 5:**
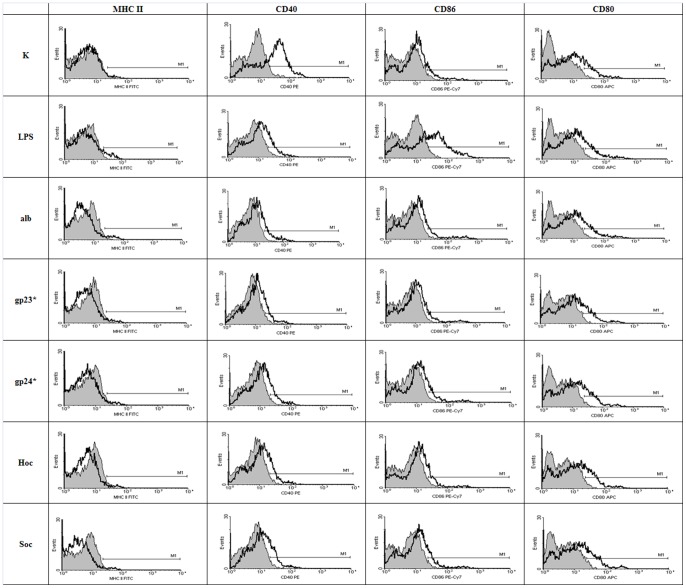
Phenotypic characteristics of BM-DCs stimulated with phage proteins. MFI (mean fluorescence intensity) was presented for the isotype control (shadowed gray histogram) vs the examined surface antigen (black). This figure presents results from one of three experiments. K – control, alb – albumin.

**Figure 6:**
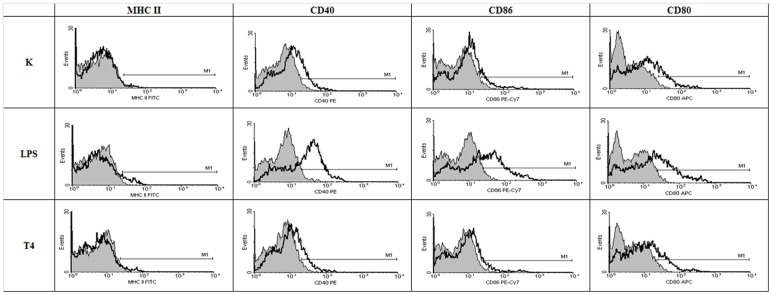
Phenotypic characteristics of BM-DCs stimulated with T4 phage. MFI (mean fluorescence intensity) was presented for the isotype control (shadowed gray histogram) vs the examined surface antigen (black). This figure presents results from one of three experiments. K – control.

**Figure 7:**
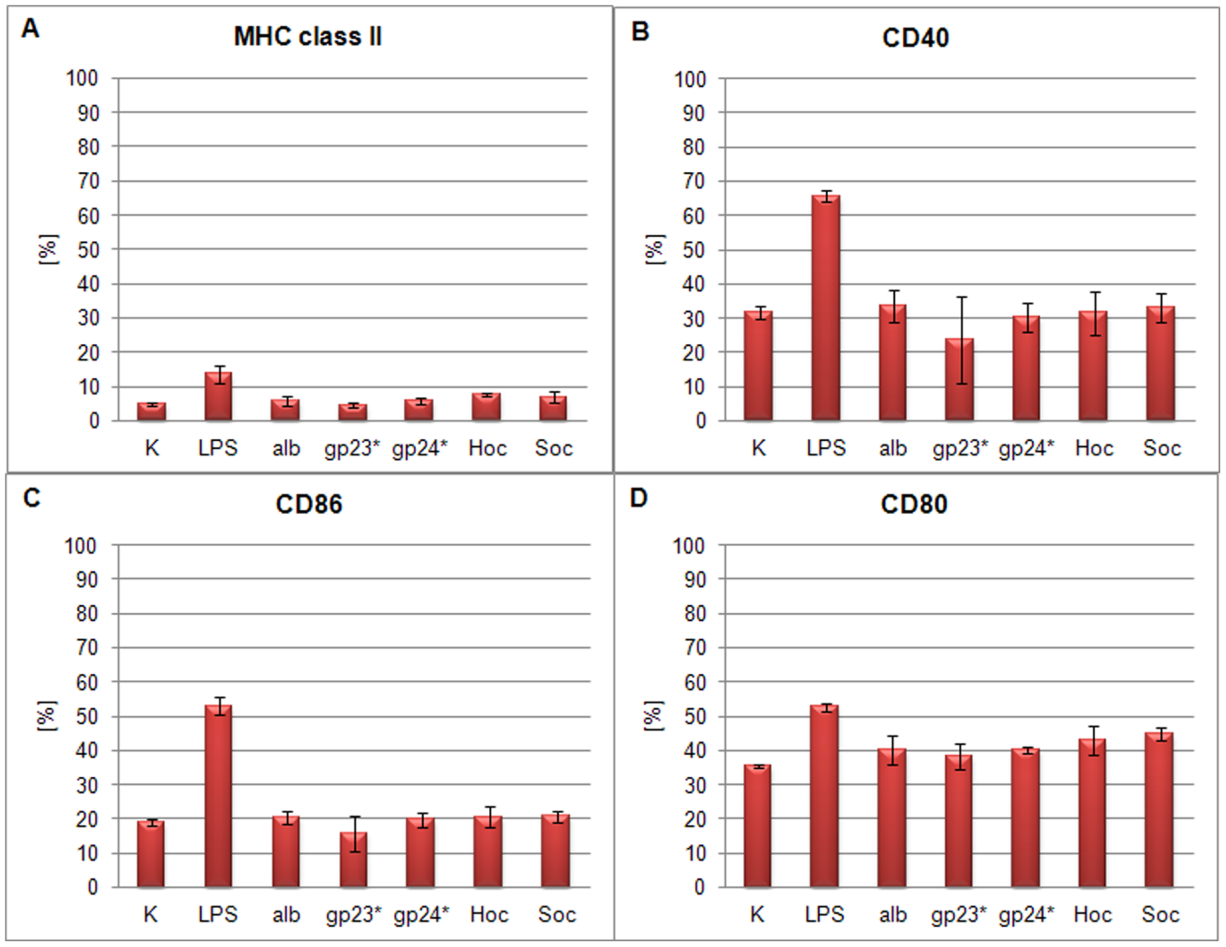
BM-DCs positive for: MHC II, CD40, CD86 and CD80 after phage proteins treatment (% of the whole cell population). Cells originated from culture of BM-DC stimulated with T4 phage proteins. As controls LPS, albumin (alb) or no-stimulated cells (K – control) were used. (A) MHC class II, (B) CD40, (C) CD86 and (D) CD80.

**Figure 8:**
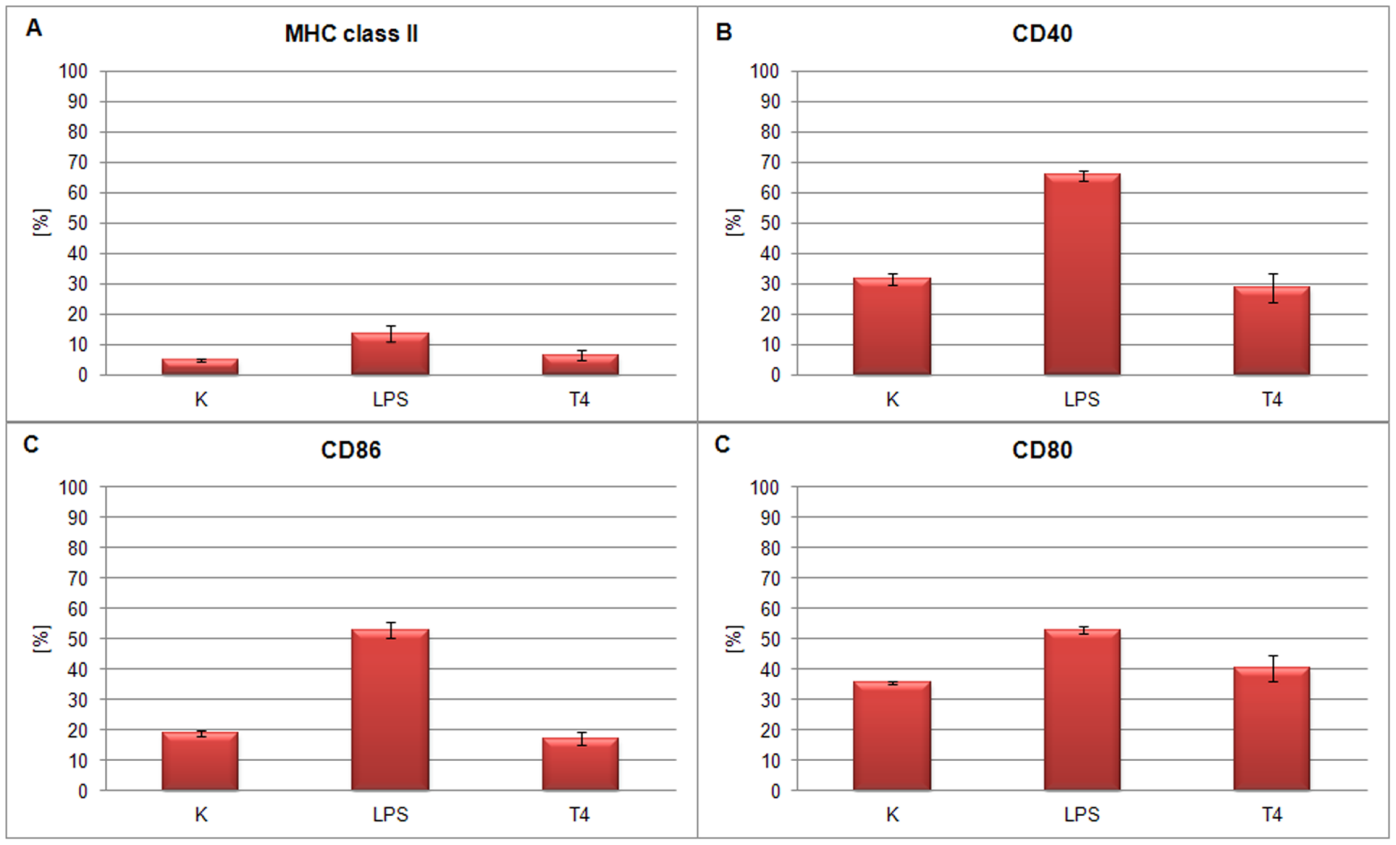
BM-DCs positive for: MHC II, CD40, CD86 and CD80 after T4 phage treatment (% of the whole cell population). Cells originated from culture of BM-DC stimulated with T4 phage. As controls LPS or no-stimulated cells (K – control) were used. (A) MHC class II, (B) CD40, (C) CD86 and (D) CD80.

BM-DCs in negative control groups were characterized by moderate expression of CD40, CD80 and CD86 co-stimulatory molecules and low expression of MHC class II molecules, whereas the LPS-stimulated BM-DCs (positive control) showed very high levels of CD40 and CD86 (Figure 5, Figure 6). After 24 h culture with T4 phage and its proteins, expression of CD40 and CD80 was not substantially changed in any group. The differences were small, insignificant and substantially lower in comparison to that induced by LPS. The level of MHC II molecule was not affected by the phage proteins nor by T4 phage and only very slightly by LPS. The lack of changes in expression of cell surface antigens was confirmed by percentage of positive cells calculated for MHC class II (Fig. 7A, Fig. 8A), CD40 (Fig. 7B, 8B), CD86 (Fig. 7C, 8C), CD80 (Fig. 7D, 8D).

### Reactive oxygen species formation in polymorphonuclear leukocytes (PMNs) or peripheral blood mononuclear cell (PBMCs)

Freshly isolated human PMNs or PBMCs were employed in the assay of reactive oxygen species generation. Measurements were accomplished by luminol-dependent chemiluminescence (CL) assay. Phorbol 12-myristate 13-acetate (PMA) and LPS, previously shown to be potent stimulators of ROS [24], [25], served as positive controls, whereas PBS or albumin were negative controls.

**Figure 9:**
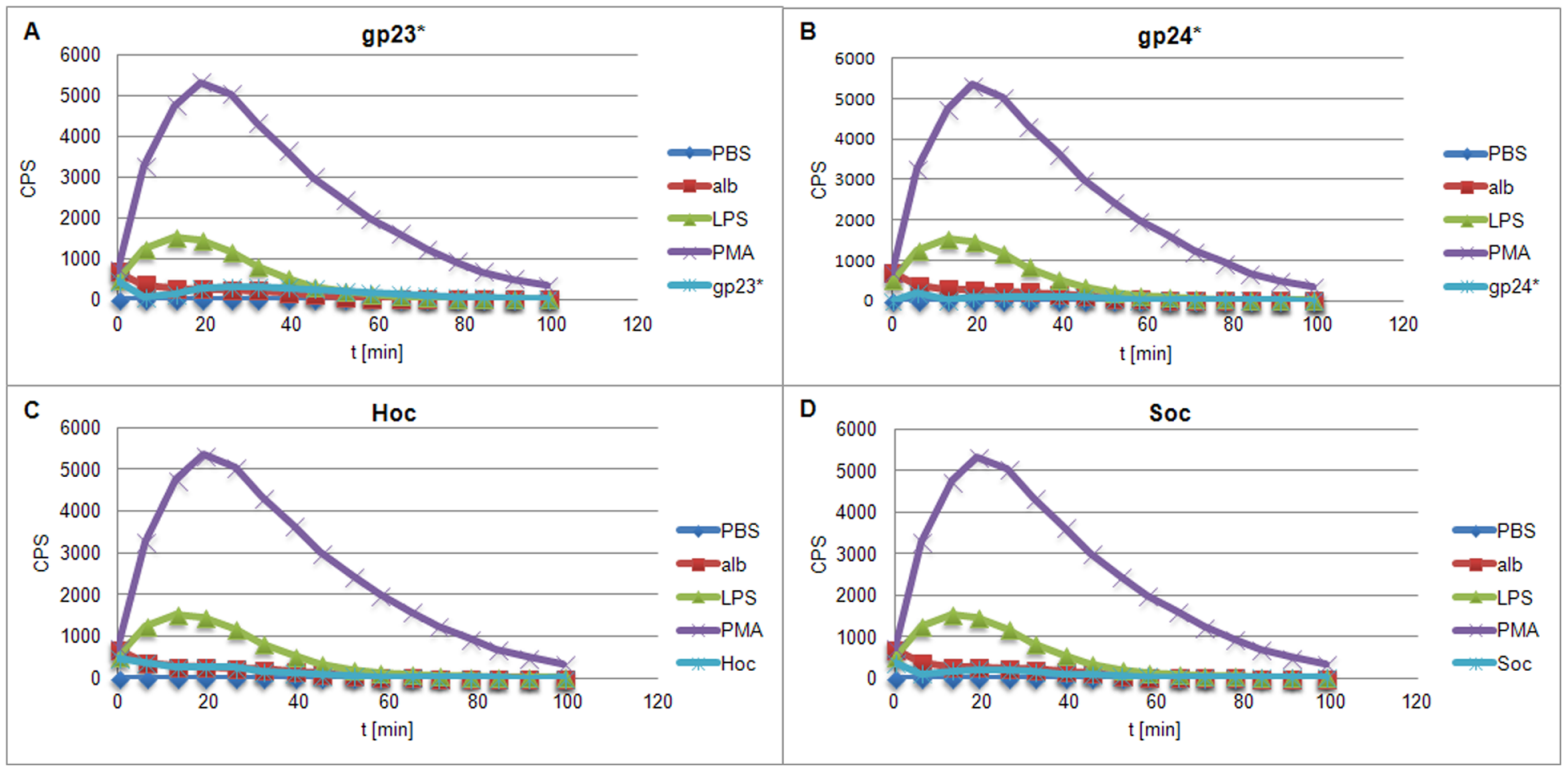
Kinetics of ROS formation by PMNs stimulated by gp23*, gp24*, Hoc, and Soc. (A) gp23*, (B) gp24*, (C) Hoc, (D) Soc. Alb – albumin.

**Figure 10:**
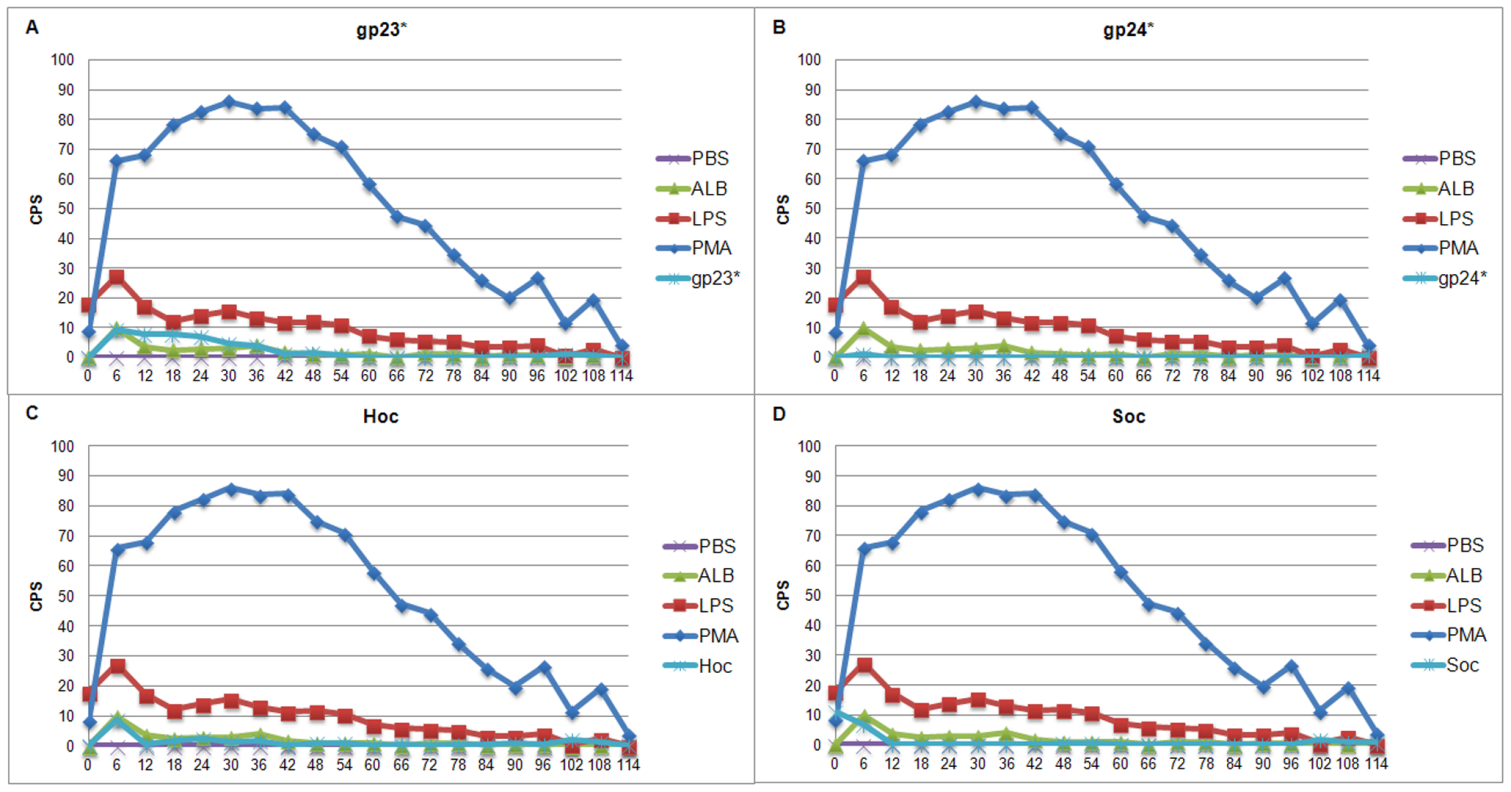
Kinetics of ROS formation by PBMCs stimulated by gp23*, gp24*, Hoc, and Soc. (A) gp23*, (B) gp24*, (C) Hoc, (D) Soc. Alb – albumin.

**Figure 11:**
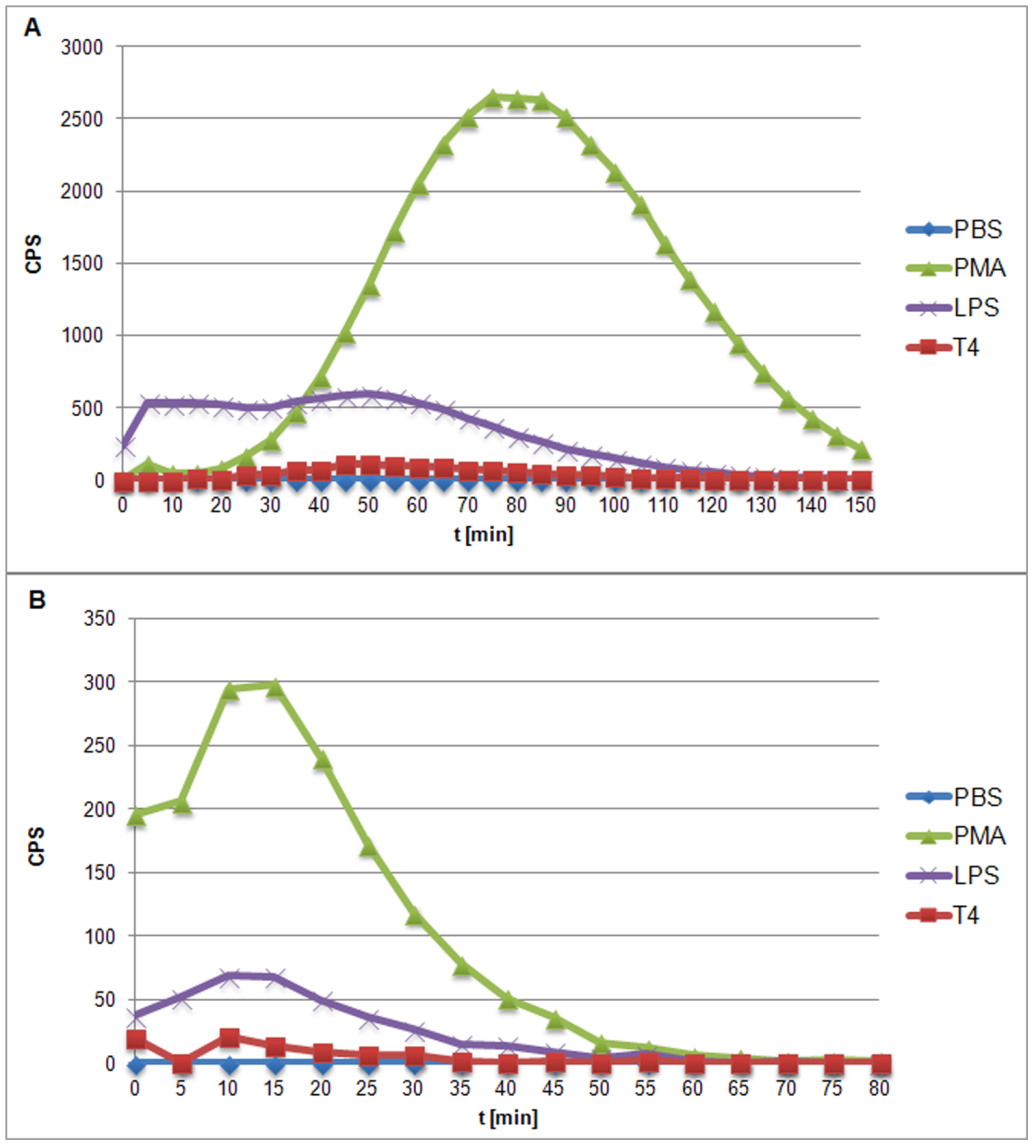
Kinetics of ROS formation by PMNs and PBMCs stimulated by T4 phage. (A) PMNs, (B) PBMCs.

As expected, PMA induced the highest level of ROS in both PMNs and PBMCs. Its strong influence on blood cells was also maintained the longest. LPS effect on ROS production was weaker than that of PMA, but still higher in comparison to the negative controls. ROS production in PMNs treated with phage proteins was at a level comparable to negative controls, significantly lower in comparison to the cells stimulated with PMA or LPS (Fig. 9). Similar tendency was observed during PBMCs treatment with phage proteins (Fig. 10). Whole phage particles also did not induce effect to ROS production as well as by PMNs (Fig. 11A) and by PBMCs (Fig. 11B). Thus supporting the conclusion that T4 phage and its head proteins are not able to stimulate major inflammatory processes.

## Discussion

Considering the well-known immune-stimulating activity of many virus and their proteins, the potential impact of bacterial viruses on inflammatory mediators in animals and in humans should be regarded in view of any medical or veterinary application of phages. Cytokines and ROS play a significant role in regulation of cell functions and in the immune response. However, their overproduction is harmful for cells and tissues, leading to dangerous damage and being a key trigger e.g. in septic shock. In this work, bacteriophage T4 and its most exposed and abundant proteins were investigated for their influence on inflammatory mediators such as cytokines, chemokines, growth or differentiation factors and on reactive oxygen species production.

According to our observations, bacteriophage T4 and its surface proteins gp23*, gp24*, Hoc and Soc did not affect cytokine or ROS production. None of the essential proinflammatory cytokines (TNF-α, IL-1, IL-6, IL-12) were activated in mice, murine dendritic cell cultures, or in human blood. Both the lack of IL-10 production, and minor changes in expression of analyzed antigens on stimulated dendritic cells could be a convincing argument for no efficient activity of these proteins. All the investigated models – throughput cytokine tests in mice *in vivo*, throughput cytokine tests in human blood, as well as *ex vivo* cultures of murine dendritic cells – gave the same results. Therefore we conclude that T4 phage and its head proteins gp23*, gp24*, Hoc and Soc do not induce massive immunological stimulation in mammals.

The present observations concern major capsid proteins of T4 phage. The T4 head is the biggest and the most exposed part of the capsid. Its surface proteins gp23*, gp24*, Hoc and Soc also represent the most numerous elements in the phage particle. However, there are other elements of the T4 phage capsid as well as other phage families that should also be investigated in future studies. Our studies constitute the first attempt to elucidate the problem and call for further investigation in the field. Here we presented a basic approach, but further studies should be extended to cytokine production during bacterial infections.

These observations are of importance for any medical or veterinary application of bacteriophages. Probably the most important is the prospect of phage therapy in antibiotic resistant infections [8]. In this case the intensity of phage influence could be very strong, since phages are usually applied in large amounts and they propagate in the infection site as long as bacteria are present. Phages have also recently been proposed as vectors for gene vaccines [26] or universal carriers for active peptides or proteins (phage display) [27]–[29] that may be applied *in vivo*. In all these applications, potential massive stimulation of inflammation by the phage proteins themselves would be a strong disadvantage and contraindication for the use of phages. The lack of immune-stimulation activity supports safety of the phage adaptation in medicine.

Phages are also the most abundant entities in the biosphere, with a special contribution of T4-like phages [30]. Our natural contact with this group, as well as with phages in general, is constant and very intensive. Phages are present in food, water, and as a natural part of the animal and human gut microbiome [31]. Therefore the issue of the potential phage impact on immunity also has significance for general understanding of bacteriophage biology and their role in ecosystems.

## Materials and Methods

### Ethics Statement

The female C57Bl6/J (6–8 weeks) mice were purchased from the Center of Experimental Medicine, Medical University of Bialystok, and kept in specific pathogen free (SPF) conditions in the Animal Breeding Centre of the Institute of Immunology and Experimental Therapy (IIET). The experiments were approved by 1st Local Committee for Experiments with the Use of Laboratory Animals, Wroclaw, Poland (no. 64/2009).

### Phages preparation

T4 phage was purchased from ATCC, Rockville, MD, USA. The material applied was highly purified phage preparation. The procedure was conducted as described [32]. Briefly, T4 phage lysates of *Escherichia coli* were purified by filtration through polysulfone membranes, gel filtration on Sepharose 4B (Millipore, Europe), celluflofine sulphate (JNC Corporation, Japan) chromatography and EndoTrap Blue ^®^ (Hyglos GmbH, Germany). The final preparations were dialyzed against PBS and filtered through 0,45 µm PES filters. Phage concentration was measured by two-layer method of Adams [33].

### Phage proteins isolation and purification

Highly purified phage proteins gp23* (a mature form of gp23 in T4 capsid), gp24* (a mature form of gp24 in T4 capsid), gphoc and gpsoc were prepared as previously described (Miernikiewicz, 2012). Briefly, genes coding the proteins were cloned using Gateway Cloning Technology to pDEST15 and pDEST24 with GST affinity tags, and expressed in a bacterial expression system. *E. coli* strain B834(DE3) F^-^ ompT hsdS_B_(r_B_^−^ m_B_^−^) gal dcm met (DE3) (EMD, Europe) was grown in Luria-Bertani Broth (LB) high salt (10 g/l of NaCl) culture medium (Sigma-Aldrich, Europe or AppliChem, Europe). Most of the proteins were co-expressed with chaperones: gpsoc with TF chaperone of *E. coli* (from pTf16 vector, TaKaRa Bio Inc., Europe), gphoc with groES+groEL of *E. coli* (from pGRO7 vector, TaKaRa Bio Inc., Europe), and gp23* with gp31 chaperone (expressed from pG31t) of T4 phage, whereas gp24* was expressed without chaperones. Gp31 replaces GroES as a co-chaperone with GroEL and is required for correct gp23* folding in the course of phage maturation. Induction was elicited by IPTG (0.2 mM) or L-arabinose (3 mM) and conducted overnight at 25°C. Harvested bacteria were resuspended in phosphate buffer (50 mM Na_2_HPO_4_, 300 mM NaCl, pH 8.0) and treated with PMSF (1 mM), lysozyme (0.5 mg/ml), low temperature −80°C (freeze-thaw method), DNase (10 µg/ml), and RNase (20 µg/ml). Soluble fractions were incubated with glutathione sorbent slurry (Glutathione Sepharose 4B, GE Healthcare Life Sciences, Europe), washed with phosphate buffer and released by proteolysis with specific protease AcTev at 10°C (5 U/ml) (Invitrogen, Life Technologies Corporation). Next, LPS was removed using EndoTrap Blue^®^ (Hyglos GmbH, Germany), gel filtration was performed by FPLC (fast protein liquid chromatography) on a Superdex 75 10/300 GL column (GE Healthcare Life Sciences, Europe) and again EndoTrap Blue^®^ was used. Bovine albumin (Sigma, Europe), which served as a control, was purified in the same way as phage proteins starting from the LPS removal step. The final preparations were dialyzed against PBS and filtered through 0.22 µm PVDF filters (Millipore, Europe). Their concentrations were determined by Lowry assay (Fermentas International Inc.).

### Determination of endotoxin activity

Endotoxin level of the purified T4 phage preparations and the proteins was assessed using the QLC-1000 Endpoint Chromogenic LAL test kit (Lonza). 50 µl of examined samples were transferred into endotoxin-free tubes. All preparations had a negative control (i.e. apyrogenic LAL water) and were prepared in duplicate. Samples along with 4 standards were kept at 37°C for 5 min. 50 µl of LAL lysate was added and incubated for 10 min at 37°C. Next 100 µl of the substrate was added, mixed gently and incubated for 8 min at 37°C. The reaction was terminated by adding the stopping reagent and mixed. 200 µl of samples were transferred to a microplate and the absorbance at 405–410 nm was measured within a 30 min period.

### Lipopolysaccharide extraction

*E. coli* were grown for 48 h at 37°C in LB medium vigorously aerated by shaking, killed with 0.5% phenol and centrifuged at 39 000 rpm using a flow centrifuge (New Brunswick Scientific, USA). The bacterial pellet was washed three times with distilled water, lyophilized, treated with 90% phenol/water (1:1), and heated to 65°C. LPS was extracted for 15 min according to Westphal and Jann [34]. The extract was cooled to 4°C, centrifuged at 3 000×g for 30 min and the water phase was collected. Distilled water was added to the remaining phenol phase and the extraction process was repeated. Both water phases were combined, dialyzed against water for 72 h (MWCO 12–14 kDa) and lyophilized. To remove nucleic acids, the resultant LPS was ultra-centrifuged (105 000×g, 6 h, repeated two times), and the LPS suspension was lyophilized again. The final sample was sonicated and diluted with PBS to the various desired concentrations.

### Mouse plasma preparation

Mice were injected intraperitoneally with 20 µg/mouse of highly purified protein preparations. Albumin (20 µg/mouse) and PBS served as two independent controls. All protein preparations had on average ≤2.1 EU/mouse of LPS. After 5.5 hours [35] murine blood was collected from the orbital plexus vein into heparinized tubes, under anesthesia. Then animals were sacrificed by cervical dislocation. The plasma was separated through two centrifugations, at 2250×g for 10 min or 5 min, and used for Mouse Cytokine Antibody Array. Two mice per group were used.

### Human plasma preparation

The whole blood was drawn from healthy volunteers into heparinized tubes and incubated at 37°C with the protein preparations (10 µg/ml). Experiments were approved by the local Commission of Bioethics, Wroclaw Medical University. The control samples were incubated with albumin (10 µg/ml) and PBS. Final concentration of LPS in all preparations was ≤0.31 EU. After 5.5 hours the plasma was separated through two centrifugations as mentioned above and used for Human Cytokine Antibody Array.

### Cytokine Antibody Array

Plasma samples were analyzed with cytokine antibody array by using either RayBio Human Cytokine Antibody Array 5 or RayBio Mouse Cytokine Antibody Array 3 (RayBiotech, Inc.) according to the manufacturers' instructions. Briefly, the membranes were blocked with 2 ml of 1X blocking buffer for 30 min and incubated overnight at 4°C with gentle shaking with 1.5 ml of 2-fold diluted plasma. Wash steps were performed as follows: 3 times with 1X Wash Buffer I and 2 times with 1X Wash Buffer II. Next, membranes were incubated for 1.5 h at room temperature (RT) with 1 ml of diluted biotin-conjugated antibodies, washed and treated with 2 ml of 1 000**-**fold diluted horseradish peroxidase-conjugated streptavidin for 2 h at RT, and again washed. The detection reaction was developed with peroxidase substrate (mix of detection buffers C and D provided by producer) incubated for 2 minutes at RT with each membrane. The signal was directly detected using a chemiluminescence imaging system. Each assay (murine or human) was repeated three times; one exemplary experiment was presented.

Density was measured applying Quantity One –4.6.9 software (Bio-Rad) according to the manufacturer's recommendation and instructions. The density background value was subtracted from each test sample value and normalized (according to the manufacturer's manual) to allow comparison of readouts from separate membranes. The formula was as follows:
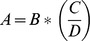
A – normalized signal intensity of particular spot B – signal intensity of particular spot C – positive signal of albumin D – positive signal of sample in particular spot.

### Generation of bone marrow-derived dendritic cells

Dendritic cells (DCs) were prepared from bone marrow isolated from the long bones of female C57BL/6 mice. Briefly, the cells were flushed from the femurs and tibias with RPMI (Gibco, Europe) medium, washed three times and re-suspended in RPMI supplemented with 10% (v/v) fetal bovine serum (FBS) (Sigma, Europe) (10% CM). The cells were cultured in 75-cm^2^ flasks at a concentration of 1×10^7^ cells per flask in 10 ml of 10% CM supplemented with 40 ng/ml recombinant murine GM-CSF (Invitrogen, Life Technologies Corporation) and 10 ng/ml recombinant murine IL-4 (Invitrogen, Life Technologies Corporation). The CM was replaced every second day. After 6 days, the BM-DC were collected, and next the cells were transferred into 12-well plates (1×10^6^ cells/ml per well) and maintained for 2 additional days in CM with 40 ng/ml GM-CSF and 5 ng/mL IL-4. For the last 24 h, stimulators (phage T4 in final concentration 5×10^8^ pfu/ml, phage proteins in final concentration of 10 μg/ml, or LPS diluted to 300 EU/ml) were added to the BM-DC cultures. Next, culture supernatants were harvested for ELISA assays and cells for surface phenotype estimation. The quality and purity of the mouse BM-DCs, after 8 day bone marrow differentiation using GM-CSF and IL-4, were estimated by the percentage of CD11c^+^ cells and assessed before each assay. BM-DC suspension containing ca. 60% CD11c^+^ cells was used for experiments.

### Flow cytometry analysis of cell-surface phenotype

BM-DCs were suspended in cold PBS containing 2.5% fetal calf serum (FCS), washed, and then incubated in a one-step test with the fluorophore-labeled monoclonal antibodies (mAbs): hamster anti-mouse APC-CD80 (BD Pharmingen, clone 16-10A1), rat anti-mouse PE-Cy7-CD86 (BD Pharmingen, clone GL1), mouse anti-mouse FITC I-A^b^ (BD Pharmingen, clone 25-9-17), rat anti-mouse RPE-CD40 (BD Pharmingen, clone 3/23), and the appropriate isotype controls: APC-labeled Hamster IgG2,k (BD Pharmingen, clone B83-3), PE-Cy7-labeled Rat IgG2a (BD Pharmingen, clone R35-95), FITC-labeled Mouse IgG2a (BD Pharmingen, clone G155-178), R-phycoerythrin (RPE)-labeled IgG2a (BD Pharmingen, clone R35-95). The cells were stained for 45 min at 4°C. The analysis was carried out using Becton Dickinson FACSFortessa apparatus with FACSDiva software.

### ELISA assay

Twenty-four hour supernatants from the particular DC cultures were collected, stored at 4°C and next analyzed by means of commercially available ELISA kits to measure the production of IL-6, TNF-α, IL-10 and IL-12 (all Becton Dickinson).

### Preparation of polymorphonuclear leukocytes (PMNs) and peripheral blood mononuclear cells (PBMCs)

PMNs and PBMCs were isolated from buffy coats of human healthy volunteer blood (experiments were approved by the local Commission of Bioethics, Wroclaw Medical University) on a double gradient of Histopaque 1077 and 1119 (Sigma, Europe) by centrifugation at 1 970 rpm for 20 min, 20°C. The granulocyte-rich layer was harvested and washed three times (5 min, 2 000 rpm, 4°C) with Hank's balanced salt solutions (HBSS). After elimination of erythrocytes by osmotic shock (1 volume of HBSS +2 volume of mQ, pH 4.65), granulocytes were resuspended in HBSS and adjusted to 1.5×10^7^ cells/ml. PBMCs-rich layer was harvested, washed in the same way as PMNs, resuspended in HBSS and adjusted to 1×10^7^ cells/ml. Cell morphology and condition were assessed by light microscope after staining with trypan.

### Determination of ROS

Reactive oxygen species generation by PMNs or PBMCs was measured using luminol-dependent chemiluminescence assay. Measurements were made using a Wallac 1420 microplate reader/luminometer (Perkin Elmer, USA). PMA and LPS served as positive controls, whereas PBS and albumin were negative controls. Each well contained 38 µl of Krebs-Ringer buffer, 25 µl of PMNs (10^6^ cells/ml) or 25 µl of PBMCs (10^6^ cells/ml), 25 µl of analyzed samples (100 µg/ml of gp23*, gp24*, Hoc, Soc, 10^9^ pfu/ml of T4) or stimulators: PMA (Applichem, Germany) or LPS (1 µM, 500 Eu/ml respectively). To control groups 25 µl of PBS or albumin (10 µg/ml) were added (all amounts are final concentrations). The reaction mixture was incubated for 15 min at 37°C. Then, 12 µl of luminol (Applichem, Germany) at a final concentration of 1 mM was added and immediately CL kinetics were measured, and recorded as counts per second (CPS). Each sample was in triplicate in all three experiments. All measurements were carried out at 37°C for 80–150 min, and recorded for 10 s every 5–6 min. The mean value of the PBS control was subtracted from all obtained data. The assay was repeated three times; one exemplary experiment was presented.

### Statistical analysis

For each of the cytokines the level of the protein signal (gp23*, gp24*, Hoc, Soc) was compared with the level of the control (PBS, albumin). The comparison involved nonparametric tests such as the sign test and Wilcoxon signed rank test.
